# Deferring surgical treatment of ambiguous genitalia into adolescence in girls with 21-hydroxylase deficiency: a feasibility study

**DOI:** 10.1186/s13633-016-0040-8

**Published:** 2017-01-28

**Authors:** Pierre Bougnères, Claire Bouvattier, Maryse Cartigny, Lina Michala

**Affiliations:** 1 Pediatric Endocrinology, Bicêtre Hospital, Paris South University, 78 Avenue du Général Leclerc, Kremin Bicêtre, 94270 Paris, France; 2 Pediatric Endocrinology, Jeanne de Flandre Hospital, Lille University, Lille, France; 3 First Department of Obstetrics and Gynaecology, University of Athens, Alexandra Hospital, Athens, Greece

**Keywords:** 21-hydroxylase deficiency, Ambiguous genitalia, Congenital adrenal hyperplasia, Genital surgery

## Abstract

**Background:**

Genital surgery in Disorders of Sex Development (DSD) has been an area of debate over the past 20 years. Emerging scientific evidence in the late 1990s defied the then routine practice to surgically align genitalia to the sex of rearing, as early as possible. However, despite multitude of data showing detrimental effects to genital sensation and sexuality, few patients born with ambiguous genitalia have remained unoperated into adolescence.

**Methods:**

We followed up girls with 21 hydroxylase deficiency (21- OHD) in genital morphology during childhood and acceptability among patients and parents of such an approach.

**Results:**

Preliminary results from 7 children, aged 1–8 years (median 4.5 years), suggest that it is acceptable among patients and families to defer genital operation in 21-OHD. All patients had a Prader stage III and above. Median clitoral length at birth was 24 mm (20-28 mm) and had diminished to a median of 9 mm (5-15 mm) at their last visit. Height and weight have remained strictly normal in all patients. So far girls and their parents have not expressed significant concerns regarding genital ambiguity.

**Conclusions:**

With this encouraging data at hand, we propose to formally address levels of anxiety, adaptation and quality of life during childhood, with an ultimate goal to assess long term satisfaction and effects on sexuality through deferring genital surgery for adolescence.

## Background

Surgical management in Disorders of Sex Development (DSD) remains an area of controversy, even following the 2005 Chicago consensus, which attempted to delineate treatment guidelines and advocated a more cautious approach to genital surgery [8]. For years, the norm in DSD, was to align genitalia with the assigned gender as early as possible. Supporters of early surgery have based their practice on a need to reinforce sex of rearing, while relieving parental tension regarding the ambiguity of the genitalia [13]. Early surgery, when assessed in the immediate postoperative period was thought to provide ideal cosmetic and anatomical results, with the additional benefit that a procedure performed early enough in infancy would be forgotten by the patient.

It was not until the 1990s that this practice was challenged by patients themselves, who came forward with significant problems in adulthood, including anatomical difficulties in penetrative sexual intercourse and decreased genital sensation or ability to reach orgasm [15].

Multiple scientific voices have joined those of patients and support groups to question long term results of early genital surgery [5, 6, 12, 14]. Decreased genital sensitivity and need for further surgery in adulthood, as well as poor cosmetic results in the long run have been proven by studies in multiple institutions, suggesting dissatisfaction in a significant proportion of patients irrespective of geographical provenance [2, 6, 11, 14].

Despite concerns, inertia has perpetuated the practice of early genital surgery into the present, and, to date, there have been no series of patients left unoperated until adolescence or adulthood so as to form a basis for comparison. Current practice still favours early genital surgery, usually based on nebulous and unproven scientific facts, as shown by national statistics [7], audits of practice among pediatric surgeons [16], and the paucity of data on unoperated patients in series of 21-OHD patients. Nevertheless, the handful of DSD patients that have remained unoperated, seem to have similar genital sensation to normal controls, with numbers however remaining too small to draw firm conclusions on [4]. With evidence on long term outcomes lacking, it is impossible to formulate a well-supported scientific argument for deferring genital operation.

## Methods

During the last decade, our institution, a tertiary referral centre for complex paediatric endocrinological disorders, has had a policy of offering unbiased information regarding the pros and cons of genital operation to all parents of children born with 21- hydroxylase deficiency and Prader III-IV stages. Parents are given the opportunity to discuss the care of their child with a paediatric endocrinologist, a gynaecologist with expertise in paediatric and adolescent care and if requested, a paediatric urologist. The child and parents are followed at regular intervals irrespective of whether they have opted for surgery or not. During visits, we assess the child’s behaviour, her interaction with her parents, evaluate for the presence of parental anxiety and note down any evidence of disruption to everyday activities due to the ambiguous appearance of the genitalia, urinary symptoms or clitoral erections.

## Results

We are currently following seven 21-OHD girls with a median age of 4.5 years (range 1–8 years) whose operation has been deferred.

The first girl, named GK, was born 8 years ago. She was left unoperated following a decision from the parents and PB. After observing the girl during one year, it was clear that the length of the clitoris could be minimized with 50 mg hydrocortisone/m2 daily in four divided doses, bring serum testosterone and delta-4-androstenedione levels to under 0.02 ng/ml. Over the following years, GK’s parents did not express any concern regarding the psychological or physical well-being of their child, despite episodic clitoral erections.

**Fig. 1:**
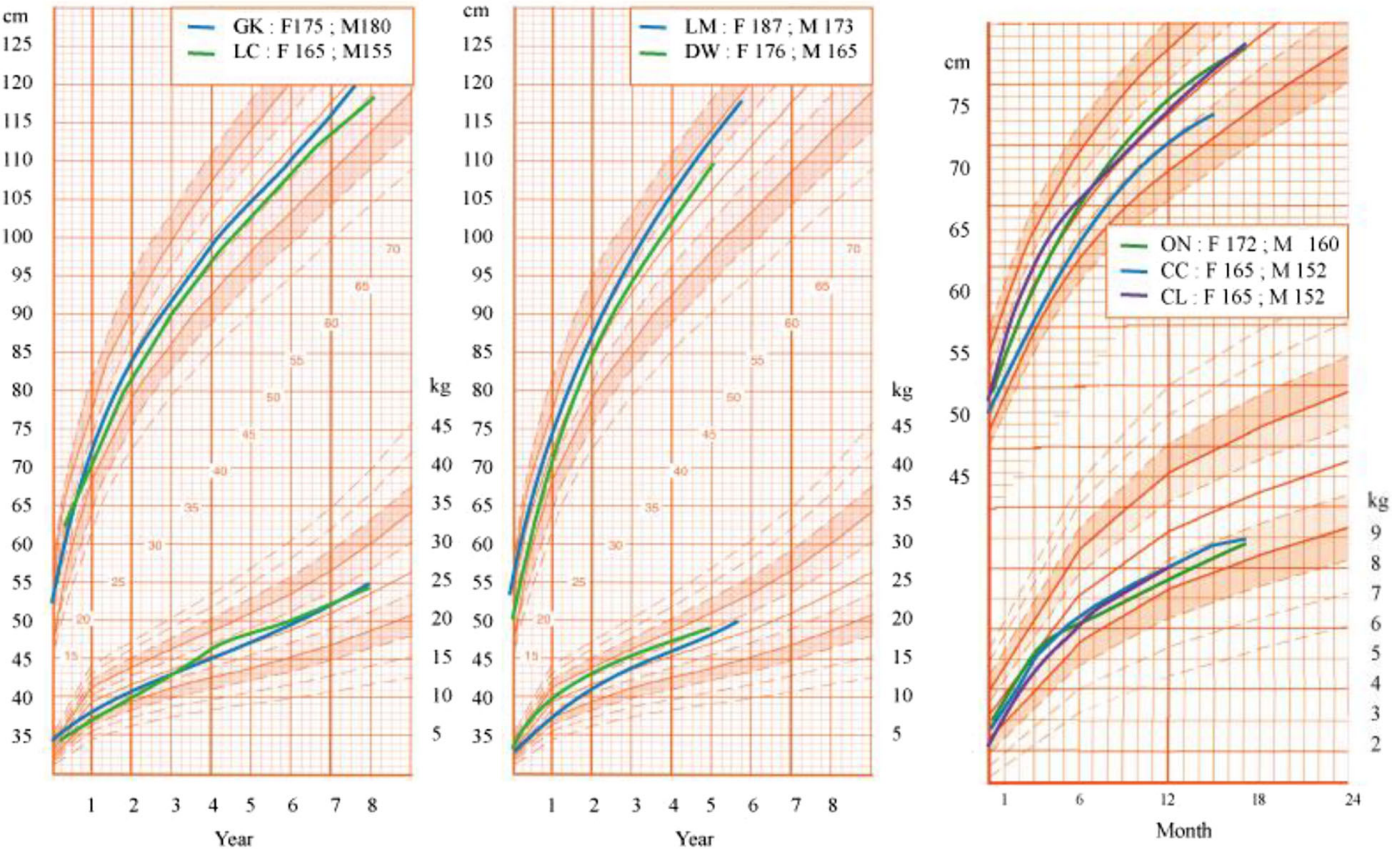
Growth curve for height and weight of 4 girls (GK, LC, DW, LM) and 3 babies. Heights of father (F) and mother (M)

**Fig. 2:**
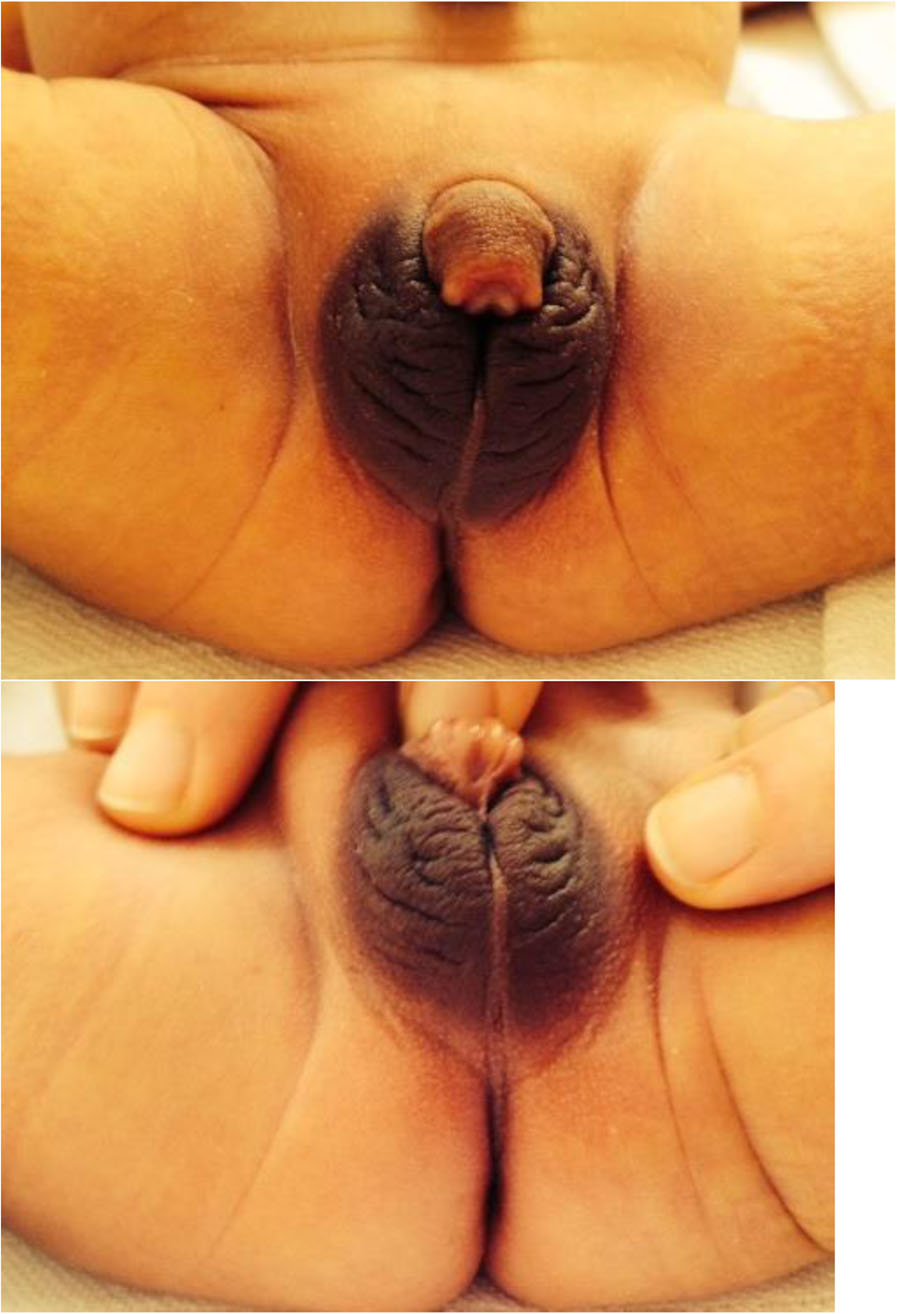
Aspects of external genitalia of ON at birth

**Fig. 3:**
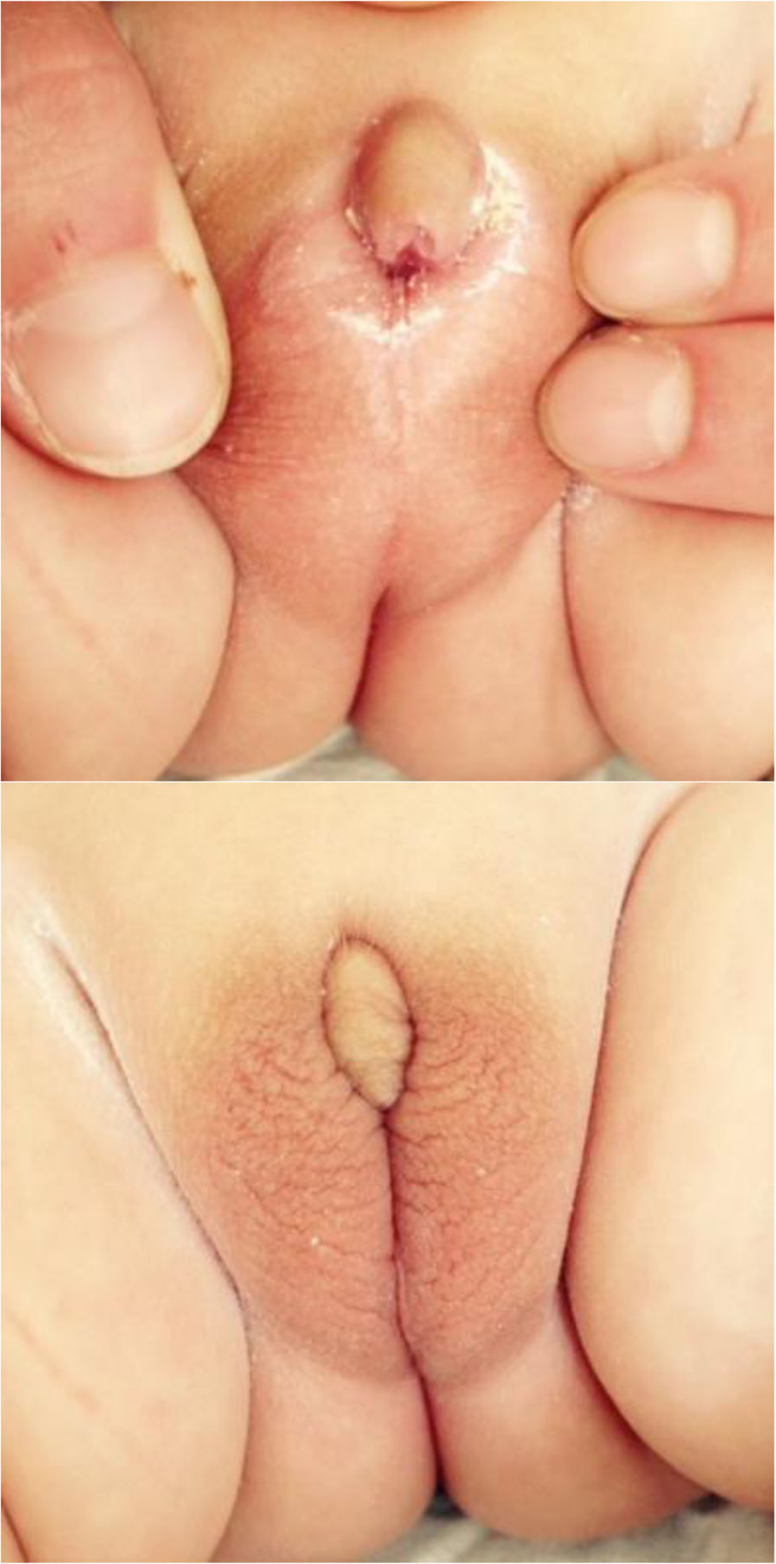
External genitalia at two months of age

Since GK’s experience was encouraging, this pilot 2-year observation was shared with CB and MC who used it to inform other parents. From 2010 to 2015, CB and MC have been following 6 more girls with 21-OHD, born with Prader III-IV stages, who were left unoperated. These girls are followed up closely, with regards to their growth pattern, the degree of change in clitoral size and their psycho-developmental adjustment. So far all girls and their parents have not expressed significant concerns regarding genital ambiguity, despite episodic clitoral erections. There have been no cases of urinary track infections. Median clitoral length in this group of patients at birth was 25 mm (range 20-28 mm), whereas at their last visit median length was 9 mm (range 5-15 mm *p* < 0.001, Wilcoxon signed rank test). Growth rate, height and weight have remained strictly normal in all patients (Fig. 1). The main characteristics of the seven girls are depicted in Table 1. Figures 2 and 3 shows the results of the treatment at age 1 month in girl ON followed by MC. Treatment in all girls was started at a dosage of 50 mg/m2 daily in four divided doses, a higher dosage than usually recommended, for the first year of life and 50 μg fludrocortisone administered twice daily, and 2gr NaCL daily divided in four doses.. Thereafter, the daily dosage of hydrocortisone was gradually reduced to an average of 40 mg/m2 for the second year of life and 10-25 mg/m2 from the third year of life onwards. The doses of hydrocortisone that we used are closer to the Japanese [9] than to the European or American guidelines [10].

**Table 1:** Main characteristics of the 7 girls with 21-OHD left unoperated in the current feasibility study

	Prenatal diagnosis	Prader stage at birth	Clitoris length at birth (mm)	17OHP diagnosis ng/ml	T at diagnosis (ng/ml)	Age at last examination (yrs)	Clitoris length at last examination (mm)	Height at last examination^a^ (SD)
GK	yes	3-4	25	257	8	8	15	+0.2
LC	yes	3-4	20	119	9.8	7	7	−0.3
LM	no	3-4	20	414	14	5.5	5	+0.3
DW	yes	3-4	20	150	9	4.5	5	+0.4
CL	no	3-4	25	49	14	1	10	+0.4
CC	no	3-4	25	14	6.5	1	15	0
ON	yes	4	28	230	14.8	1	9	0

^a^SD departure from height for age calculated from mid-parental target height

## Discussion

The audit of our small series demonstrates that, at least in childhood, and with appropriate medical care and psychological support, it is possible to defer genital surgery. Whilst so far no major concerns have been reported from patients and their families, it remains unclear whether patients will continue to accept their diversity as they grow.

Clitoromegaly may be obvious enough to peers and may become the element and basis of bullying. One would have to put this in context of the current vogue among ‘normal’ women (with no genital ambiguity) to readily explore their genital area and opt for plastic surgery to their normal labia minora or clitoral prepuce, in order to diminish tissue that protrudes in the genital region [3]. In this current trend, a girl growing with clitoromegaly and ambiguous genitalia should be under frequent psychological support to enable better understanding of the condition and reinforce strategies to cope with being diverse.

Nevertheless, the degree of clitoromegaly may not be accurately assessed at birth as the newborn’s genitals are under the full influence of adrenal androgens and maternal oestrogens. Although clitoromegaly is not reversible, anecdotal reports and our experience suggest that the clitoris could decrease in size as higher doses of glucocorticoid and mineralocorticoid replacement are instituted for the first year of life. Furthermore, as the child grows the relative larger size of the clitoris might become less evident.

A personalized regimen of glucocorticoids needs to be defined within the therapeutic window, maintaining complete suppression of androstenedione and testosterone production and avoiding the unwanted effects of chronic hypercorticism. Individualized dosage and daily administration timing should be defined pragmatically in each patient instead of a per kg dogmatic prescription expected to fit all cases.

Compliance to medication is a reported concern in 21-OHD, particularly when adolescents start to be more rebellious and independent, omitting dosages or resisting and resenting treatment all together. This explains why a proportion of patients operated in childhood may need revisions to the clitoris in adolescence because of regrowth of tissue in puberty, due to a resurgence of androgens. With this in mind a stricter control with more frequent visits and better psychological support would be required in adolescence [1].

When the girl reaches adolescence, an examination under anaesthesia, a cystoscopy and vaginoplasty will allow for an accurate evaluation of the girl’s genitalia, including measuring the width and length of the clitoris and assessing the distance of the urethral and vaginal confluence to the perineum and the caliber of the vagina. This examination is usually organized on a day surgery basis and can easily be accommodated around school commitments, so as to interfere to a minimum with the lifestyle of the girl.

A vaginoplasty performed in adolescence is technically similar to the one performed in infancy. Advantages in adolescence are the larger caliber of the proximal vagina, allowing for a better end result at the level of anastomosis. Certainly the presence of oestrogens should allow for easier tissue plane identification and postoperative healing. Further to these technical advantages, an adolescent is in a better position to perform postoperative vaginal dilation, which will be required to avoid the formation of strictures, a complication often reported in the literature in as high as 50% of patients having had a vaginoplasty in childhood [2, 5, 6, 11].

Irrespective of potential technical benefits of performing surgery later in life, a major ethical advantage stems from the fact that the patient herself can be involved in the decision to proceed or not with an operation to the clitoris, taking into account the implications of surgery, the benefits to appearance and the possible risks to genital sensation and sexual function. It is perceived that a number of patients may opt against surgery all together as they grow, either because of a decreased size of the clitoris (relative or true) or because of a concern of effects of surgery on genital sensitivity. In either case it can be her choice to proceed to a clitoral reduction, even if the parent or guardian still has the legal responsibility for the operation.

We will collect preliminary results over the next few years, regarding concerns stemming from the ambiguity of the genitalia during childhood. However, the final outcome of a study should be long term adjustment, both with regards to sexuality and quality of life. It is obvious that these results will be available fifteen to twenty years from now, when current infants with 21-OHD will be entering adolescence and adulthood. Development of a prospective study with long follow up needs planning not only with regards to a well-designed protocol and measures to decrease dropout rates and patients lost to follow up. We also need to provide continuity of care within our service, to the next generation of researchers and clinicians, the current trainees or young specialists that will be gathering and analyzing results in the future.

## Conclusions

Preliminary results in 7 young girls now aged 1–8 years affected with 21-OHD and born with Prader III-IV stages suggest that deferring genital operation is acceptable among patients and families. A careful medical treatment allowed the decrease of median clitoral length from 24 mm at birth to 9 mm at their last visit. So far girls and their parents have not expressed significant concerns regarding genital ambiguity.

With these encouraging data at hand, we propose to formally address levels of anxiety, adaptation and quality of life during childhood, with an ultimate goal to assess long- term satisfaction and effects on sexuality through deferring genital surgery for adolescence. These observations may pave the way for a new management of the disease in a subset of patients.

## Abbreviations

17-OH- P: 17-hydroxyprogesterone
21-OH-D: 21- hydroxylase deficiency
CAH: Congenital adrenal hyperplasia
DSD: Disorder of sex development
